# The genome sequence of the Barred Chestnut moth, *Diarsia dahlii* (Hübner, 1813)

**DOI:** 10.12688/wellcomeopenres.22587.1

**Published:** 2024-07-05

**Authors:** David C. Lees, Natural History Museum Genome Acquisition Lab, Darwin Tree of Life Barcoding collective, Wellcome Sanger Institute Tree of Life Management, Samples and Laboratory team, Wellcome Sanger Institute Scientific Operations: Sequencing Operations, Wellcome Sanger Institute Tree of Life Core Informatics team, Tree of Life Core Informatics collective, Darwin Tree of Life Consortium

**Affiliations:** 1 Natural History Museum, London, England, UK

**Keywords:** Diarsia dahlii, the Barred Chestnut moth, genome sequence, chromosomal, Lepidoptera

## Abstract

We present a genome assembly from an individual female *Diarsia dahlii* (the Barred Chestnut; Arthropoda; Insecta; Lepidoptera; Noctuidae). The genome sequence is 683.0 megabases in span. Most of the assembly is scaffolded into 32 chromosomal pseudomolecules, including the Z and W sex chromosomes. The mitochondrial genome has also been assembled and is 15.36 kilobases in length. Gene annotation of this assembly on Ensembl identified 13,177 protein coding genes.

## Species taxonomy

Eukaryota; Opisthokonta; Metazoa; Eumetazoa; Bilateria; Protostomia; Ecdysozoa; Panarthropoda; Arthropoda; Mandibulata; Pancrustacea; Hexapoda; Insecta; Dicondylia; Pterygota; Neoptera; Endopterygota; Amphiesmenoptera; Lepidoptera; Glossata; Neolepidoptera; Heteroneura; Ditrysia; Obtectomera; Noctuoidea; Noctuidae; *Diarsia*; *Diarsia dahlii* (Hübner, 1813) (NCBI:txid1804828).

## Background

*Diarsia dahlii* (Hübner, 1813) also known as the Barred Chestnut ( Waring *et al.*, 2017), is a moth in the family Noctuidae with a wingspan of 32–44 mm and 15–18 mm forewing length ( Bretherton *et al.*, 1979; Waring *et al.*, 2017). It is the type species of *Diarsia* Hübner, [1821] ( Varga & Ronkay, 2007). The moth is pale brownish or reddish with paler yellow reniform and orbicular stigmata (the latter subtended by a black dot) and contrasting slightly darker or greyish transverse fasciae or ‘bars’, especially towards the termen; compared to related species of *Diarsia* (‘Clays’) the forewing costa tends to be more rounded, and the species is more strongly sexually dimorphic (females more reddish in England, more purplish-blackish in Scotland, the latter confusable with *D. brunnea* ([Denis & Schiffermüller], 1775) ( Bretherton *et al.*, 1979; Waring *et al.*, 2017).

*D. dahlii* is univoltine, flying from mid-July to late September with no sign of a recent shift in phenology ( Randle *et al.*, 2019), preferring woodlands, moorlands and heaths on acid soils in Britain and Ireland, where it is widespread but localised, much more so in Ireland and the south of England (e.g. woodland on greensand Surrey). It is also widespread but rather localised in Western Europe, not in the Iberian Peninsula, but much commoner in Scandinavia including the far north, with scattered records through Asia as far east as Kamchatka and Japan ( GBIF Secretariat, 2024).

In Britain and Ireland, the Barred Chestnut has declined markedly in distribution especially in the south, gone from many sites pre-1970 (with a long-term distribution decline from 1970–2016 of 30%), but the overall abundance has increased by 156% ( Randle *et al.* 2019).

The rounded pale whitish egg is often laid in batches and the nocturnally active larva (see e.g. Lepiforum, 2024) feeds from October to May on woody (including sallows and birches) and herbaceous (e.g. docks *Rumex* spp.) plants and overwinters; fullfed it is c. 34 mm long ( Bretherton *et al.*, 1979). The shiny brown pupa is enclosed in a loose cocoon in the ground. The nocturnal adult nectars on flowers such as *Calluna vulgaris* (L.) Hull. and wood sage *Teucrium scorodonia* L. ( Bretherton *et al.*, 1979, Lepiforum, 2024).

The mitogenome from the genomic assembly (OX459221.1) (NHMUK014451629) is at least 0.15% divergent from other DNA barcode records from England, Scotland and Norway on BOLD (11/04/2024), belonging to the BIN cluster BOLD:AAE0664, whereas it is 2.01–2.61% divergent from others in BIN BOLD:ABX6544 from Finland, Russia and Bavaria. Only one BIN is so far known in Britain, which is just 1.92% divergent from BOLD:ABX6544 (a specimen from Finland identified as *D. brunnea* but representing the other BIN of *D. dahlii*). Members of the supposed subspecies *D. dahlii tibetica* Boursin, 1954 from China and *D. dahlii nana* (Staudinger, 1892) ( Varga & Ronkay, 2007) have not yet been DNA barcoded. *D. dahlii* belongs to the *dahlii* morphological species group that includes also *D. protodahlii* Varga & Ronkay, 2007. The Nearctic *D. esurialis* (Grote, 1881) (BOLD:ABX6710) is 2.67% pairwise divergent from the haplotype from the genomic assembly; the latter species belongs to a Nearctic species group comprising also *D. calgary* (Smith, 1898) ( Varga & Ronkay, 2007).

The genome will be helpful in exploring sister relationships in *Diarsia* and examining any genomic or biological correlates of the two mitochondrial BIN clusters.

## Genome sequence report

**Figure 1.**
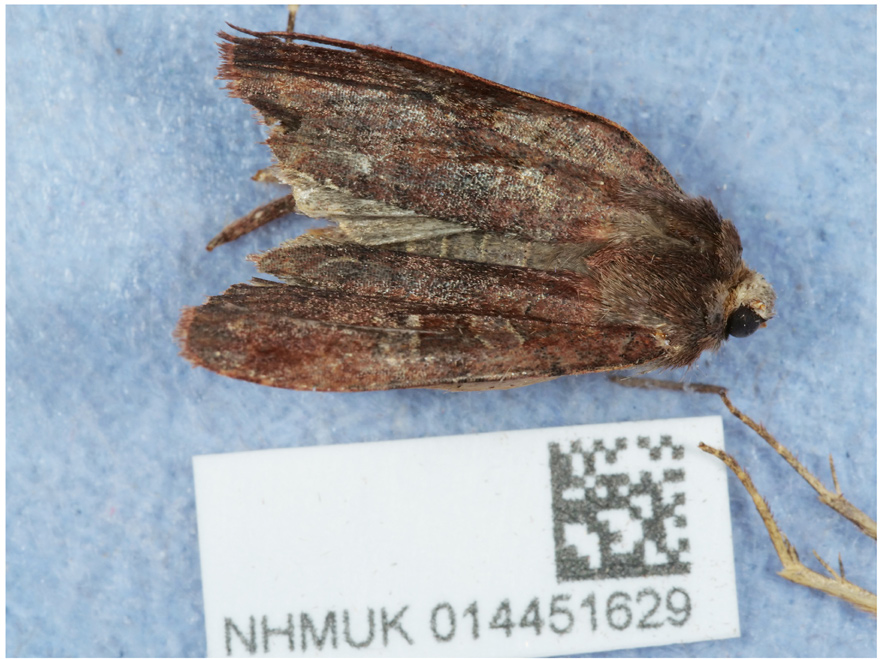
Photograph of the *Diarsia dahlii* (ilDiaDahl1) specimen used for genome sequencing.

The genome was sequenced from a female *Diarsia dahlii* ( Figure 1) collected from United Beinn Eighe National Nature Reserve, Scotland, UK (57.63, –5.35). A total of 34-fold coverage in Pacific Biosciences single-molecule HiFi long reads was generated. Primary assembly contigs were scaffolded with chromosome conformation Hi-C data. Manual assembly curation corrected 35 missing joins or mis-joins and removed 8 haplotypic duplications, reducing the assembly length by 2.20% and the scaffold number by 24.49%, and increasing the scaffold N50 by 0.20%.

**Figure 2.**
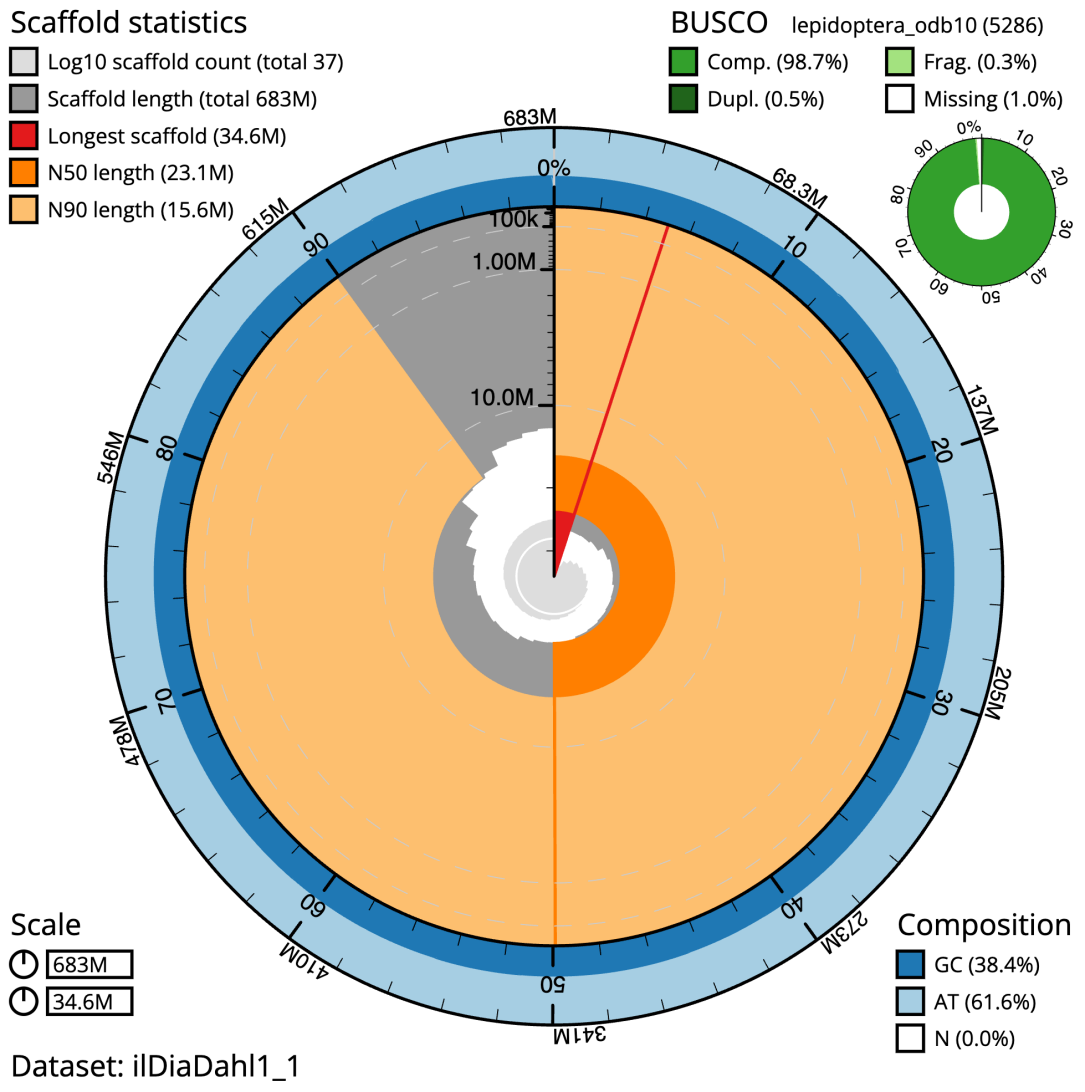
Genome assembly of *Diarsia dahlii*, ilDiaDahl1.1: metrics. The BlobToolKit snail plot shows N50 metrics and BUSCO gene completeness. The main plot is divided into 1,000 size-ordered bins around the circumference with each bin representing 0.1% of the 682,983,932 bp assembly. The distribution of scaffold lengths is shown in dark grey with the plot radius scaled to the longest scaffold present in the assembly (34,578,549 bp, shown in red). Orange and pale-orange arcs show the N50 and N90 scaffold lengths (23,141,363 and 15,636,754 bp), respectively. The pale grey spiral shows the cumulative scaffold count on a log scale with white scale lines showing successive orders of magnitude. The blue and pale-blue area around the outside of the plot shows the distribution of GC, AT and N percentages in the same bins as the inner plot. A summary of complete, fragmented, duplicated and missing BUSCO genes in the lepidoptera_odb10 set is shown in the top right. An interactive version of this figure is available at https://blobtoolkit.genomehubs.org/view/ilDiaDahl1_1/dataset/ilDiaDahl1_1/snail.

**Figure 3.**
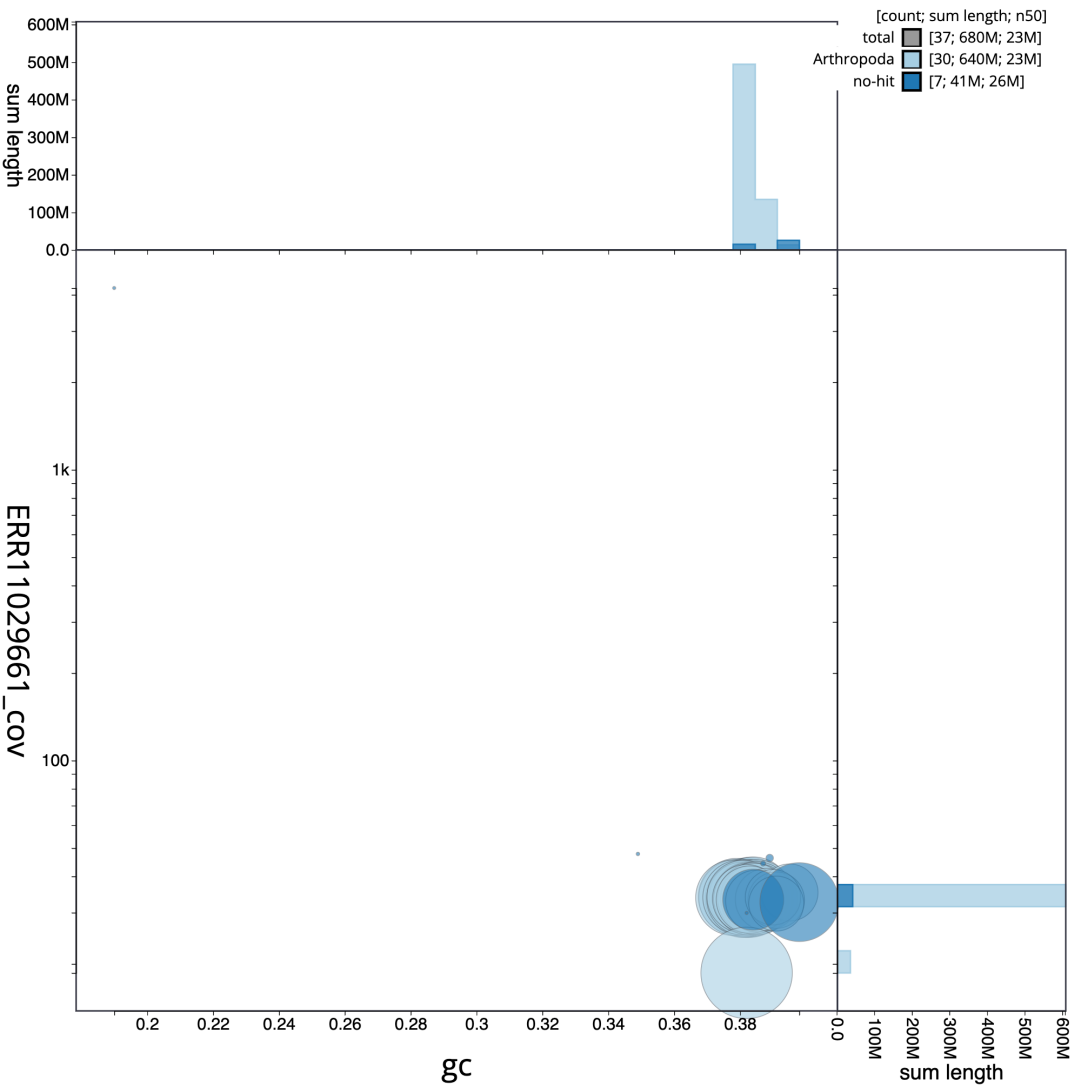
Genome assembly of *Diarsia dahlii*, ilDiaDahl1.1: BlobToolKit GC-coverage plot. Sequences are coloured by phylum. Circles are sized in proportion to sequence length. Histograms show the distribution of sequence length sum along each axis. An interactive version of this figure is available at https://blobtoolkit.genomehubs.org/view/ilDiaDahl1_1/dataset/ilDiaDahl1_1/blob.

**Figure 4.**
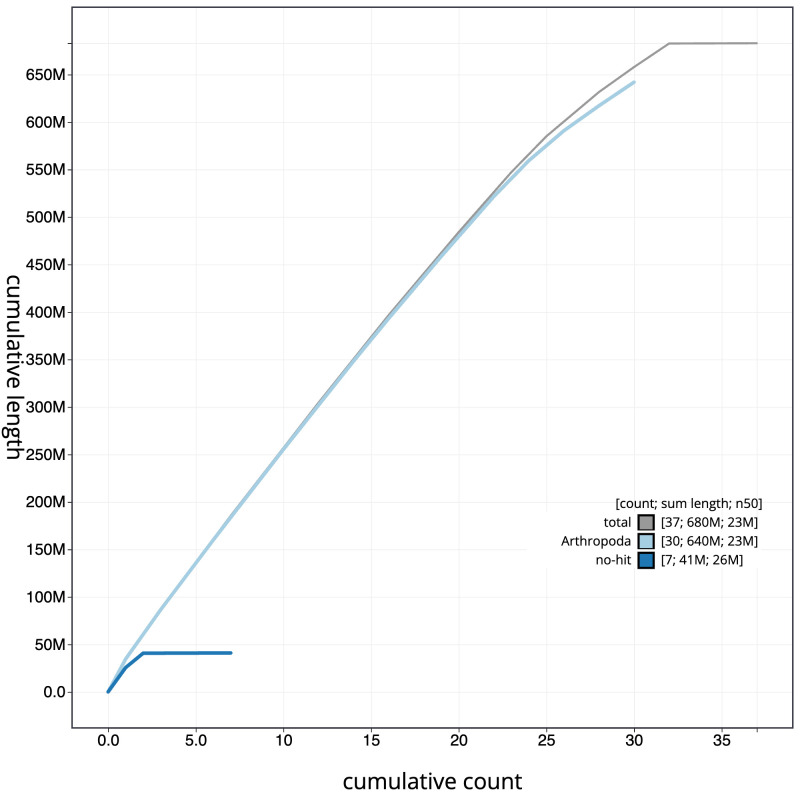
Genome assembly of *Diarsia dahlii*, ilDiaDahl1.1: BlobToolKit cumulative sequence plot. The grey line shows cumulative length for all sequences. Coloured lines show cumulative lengths of sequences assigned to each phylum using the buscogenes taxrule. An interactive version of this figure is available at https://blobtoolkit.genomehubs.org/view/ilDiaDahl1_1/dataset/ilDiaDahl1_1/cumulative.

**Figure 5.**
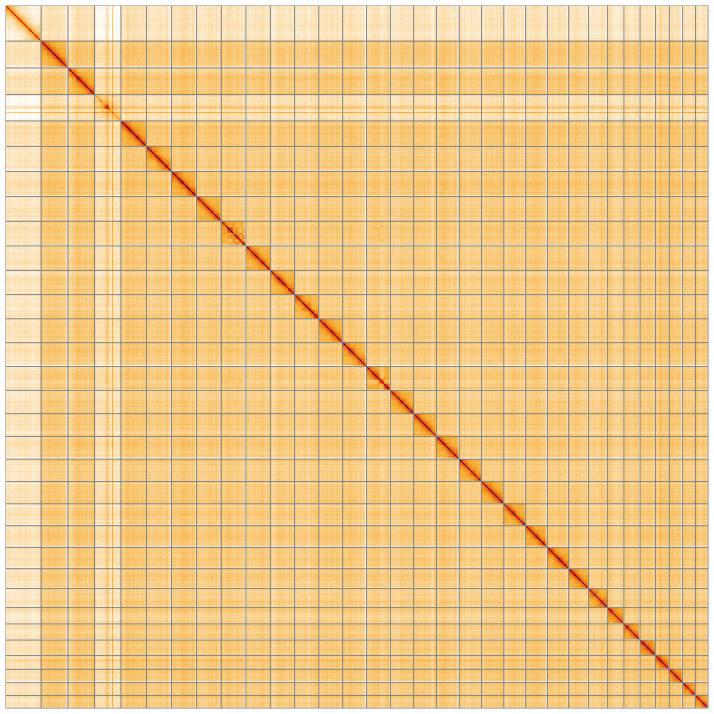
Genome assembly of *Diarsia dahlii*, ilDiaDahl1.1: Hi-C contact map of the ilDiaDahl1.1 assembly, visualised using HiGlass. Chromosomes are shown in order of size from left to right and top to bottom. An interactive version of this figure may be viewed at https://genome-note-higlass.tol.sanger.ac.uk/l/?d=cC69O4MsTNqCZ3Q0Wd-WZQ.

The final assembly has a total length of 683.0 Mb in 36 sequence scaffolds with a scaffold N50 of 23.1 Mb ( Table 1). The snail plot in Figure 2 provides a summary of the assembly statistics, while the distribution of assembly scaffolds on GC proportion and coverage is shown in Figure 3. The cumulative assembly plot in Figure 4 shows curves for subsets of scaffolds assigned to different phyla. Most (99.99%) of the assembly sequence was assigned to 32 chromosomal-level scaffolds, representing 30 autosomes and the Z and W sex chromosomes. Chromosome-scale scaffolds confirmed by the Hi-C data are named in order of size ( Figure 5; Table 2). The Z chromosome was identified by coverage and alignment to *Diarsia rubi* (GCA_932274075.1) ( Boyes *et al.*, 2023), and the W chromosome identified by read coverage. While not fully phased, the assembly deposited is of one haplotype. Contigs corresponding to the second haplotype have also been deposited. The mitochondrial genome was also assembled and can be found as a contig within the multifasta file of the genome submission.

**Table 1.** Genome data for *Diarsia dahlii*, ilDiaDahl1.1.

Project accession data		
Assembly identifier	ilDiaDahl1.1	
Species	*Diarsia dahlii*	
Specimen	ilDiaDahl1	
NCBI taxonomy ID	1804828	
BioProject	PRJEB60641	
BioSample and isolate information	PacBio HiFi sequencing: ilDiaDahl1, female, head and thorax SAMEA14448499 Hi-C scaffolding: ilDiaDahl1, female: abdomen SAMEA14448498 RNA sequencing: ilDiaDahl1, female, remaining head and thorax tissue SAMEA14448497	
Assembly metrics *		*Benchmark*
Consensus quality (QV)	65.4	*≥ 50*
*k*-mer completeness	100.0%	*≥ 95%*
BUSCO **	C:98.7%[S:98.1%,D:0.5%],F:0.3%,M:1.0%,n:5,286	*C ≥ 95%*
Percentage of assembly mapped to chromosomes	99.99%	*≥ 95%*
Sex chromosomes	ZW	*localised homologous pairs*
Organelles	Mitochondrial genome: 15.36 kb	*complete single alleles*
Raw data accessions		
PacificBiosciences SEQUEL II	ERR11029661	
Hi-C Illumina	ERR11040173	
PolyA RNA-Seq Illumina	ERR11641131	
Genome assembly		
Assembly accession	GCA_949775195.1	
*Accession of alternate haplotype*	GCA_949775585.1	
Span (Mb)	683.0	
Number of contigs	111	
Contig N50 length (Mb)	10.1	
Number of scaffolds	36	
Scaffold N50 length (Mb)	23.1	
Longest scaffold (Mb)	34.58	
**Genome annotation**		
Number of protein-coding genes	13,177	
Number of non-coding genes	2,604	
Number of gene transcripts	23,666	

* Assembly metric benchmarks are adapted from column VGP-2020 of “Table 1: Proposed standards and metrics for defining genome assembly quality” from Rhie *et al.* (2021). ** BUSCO scores based on the lepidoptera_odb10 BUSCO set using version 5.3.2. C = complete [S = single copy, D = duplicated], F = fragmented, M = missing, n = number of orthologues in comparison. A full set of BUSCO scores is available at https://blobtoolkit.genomehubs.org/view/ilDiaDahl1_1/dataset/ilDiaDahl1_1/busco.

**Table 2.** Chromosomal pseudomolecules in the genome assembly of *Diarsia dahlii*, ilDiaDahl1.

INSDC accession	Chromosome	Length (Mb)	GC%
OX459190.1	1	26.04	38.0
OX459191.1	2	25.94	38.5
OX459193.1	3	24.75	38.5
OX459194.1	4	24.38	38.0
OX459195.1	5	24.32	38.5
OX459196.1	6	24.03	38.0
OX459197.1	7	23.91	38.5
OX459198.1	8	23.77	38.0
OX459199.1	9	23.6	38.0
OX459200.1	10	23.49	38.0
OX459201.1	11	23.21	38.0
OX459202.1	12	23.14	38.0
OX459203.1	13	23.1	38.5
OX459204.1	14	22.77	38.0
OX459205.1	15	22.13	38.0
OX459206.1	16	22.0	38.5
OX459207.1	17	21.72	38.0
OX459208.1	18	21.68	38.5
OX459209.1	19	21.26	38.5
OX459210.1	20	21.01	38.5
OX459211.1	21	20.78	38.5
OX459212.1	22	19.18	38.0
OX459213.1	23	18.64	38.5
OX459214.1	24	15.83	39.0
OX459215.1	25	15.64	38.5
OX459216.1	26	15.14	38.5
OX459217.1	27	13.3	39.5
OX459218.1	28	12.99	39.0
OX459219.1	29	12.52	39.0
OX459220.1	30	12.3	39.0
OX459192.1	W	25.58	40.0
OX459189.1	Z	34.58	38.0
OX459221.1	MT	0.02	19.0

The estimated Quality Value (QV) of the final assembly is 65.4 with *k*-mer completeness of 100.0%, and the assembly has a BUSCO v5.3.2 completeness of 98.7% (single = 98.1%, duplicated = 0.5%), using the lepidoptera_odb10 reference set ( *n* = 5,286).

Metadata for specimens, barcode results, spectra estimates, sequencing runs, contaminants and pre-curation assembly statistics are given at https://links.tol.sanger.ac.uk/species/1804828.

## Genome annotation report

The *Diarsia dahlii* genome assembly (GCA_949775195.1) was annotated at the European Bioinformatics Institute (EBI) on Ensembl Rapid Release. The resulting annotation includes 23,666 transcribed mRNAs from 13,177 protein-coding and 2,604 non-coding genes ( Table 1; https://rapid.ensembl.org/Diarsia_dahlii_GCA_949775195.1/Info/Index).

## Methods

### Sample acquisition and nucleic acid extraction

A female *Diarsia dahlii* (specimen ID NHMUK014451629, ToLID ilDiaDahl1) was collected from Beinn Eighe National Nature Reserve, Scotland, UK (latitude 57.63, longitude –5.35) on 2021-09-09 using a light trap. The specimen was collected and identified by David Lees (Natural History Museum) and preserved by dry freezing at –80 °C.

The workflow for high molecular weight (HMW) DNA extraction at the Wellcome Sanger Institute (WSI) includes a sequence of core procedures: sample preparation; sample homogenisation, DNA extraction, fragmentation, and clean-up. In sample preparation, the ilDiaDahl1 sample was weighed and dissected on dry ice ( Jay *et al.*, 2023). Tissue from the head and thorax was homogenised using a PowerMasher II tissue disruptor ( Denton *et al.*, 2023a).

HMW DNA was extracted in the WSI Scientific Operations core using the Automated MagAttract v2 protocol ( Oatley *et al.*, 2023). The DNA was sheared into an average fragment size of 12–20 kb in a Megaruptor 3 system with speed setting 31 ( Bates *et al.*, 2023). Sheared DNA was purified by solid-phase reversible immobilisation ( Strickland *et al.*, 2023): in brief, the method employs a 1.8X ratio of AMPure PB beads to sample to eliminate shorter fragments and concentrate the DNA. The concentration of the sheared and purified DNA was assessed using a Nanodrop spectrophotometer and Qubit Fluorometer and Qubit dsDNA High Sensitivity Assay kit. Fragment size distribution was evaluated by running the sample on the FemtoPulse system.

RNA was extracted from remaining head and thorax tissue of ilDiaDahl1 in the Tree of Life Laboratory at the WSI using the RNA Extraction: Automated MagMax™ *mir*Vana protocol ( do Amaral *et al.*, 2023). The RNA concentration was assessed using a Nanodrop spectrophotometer and a Qubit Fluorometer using the Qubit RNA Broad-Range Assay kit. Analysis of the integrity of the RNA was done using the Agilent RNA 6000 Pico Kit and Eukaryotic Total RNA assay.

Protocols developed by the WSI Tree of Life laboratory are publicly available on protocols.io ( Denton *et al.*, 2023b).

### Sequencing

Pacific Biosciences HiFi circular consensus DNA sequencing libraries were constructed according to the manufacturers’ instructions. Poly(A) RNA-Seq libraries were constructed using the NEB Ultra II RNA Library Prep kit. DNA and RNA sequencing was performed by the Scientific Operations core at the WSI on Pacific Biosciences SEQUEL II (HiFi) and Illumina NovaSeq 6000 (RNA-Seq) instruments. Hi-C data were also generated from abdomen tissue of ilDiaDahl1 using the Arima v2 kit. The Hi-C sequencing was performed using paired-end sequencing with a read length of 150 bp on the Illumina NovaSeq 6000 instrument.

### Genome assembly, curation and evaluation

Assembly was carried out with Hifiasm ( Cheng *et al.*, 2021) and haplotypic duplication was identified and removed with purge_dups ( Guan *et al.*, 2020). The assembly was then scaffolded with Hi-C data ( Rao *et al.*, 2014) using YaHS ( Zhou *et al.*, 2023). The assembly was checked for contamination and corrected as described previously ( Howe *et al.*, 2021). Manual curation was performed using HiGlass ( Kerpedjiev *et al.*, 2018) and PretextView ( Harry, 2022). The mitochondrial genome was assembled using MitoHiFi ( Uliano-Silva *et al.*, 2023), which runs MitoFinder ( Allio *et al.*, 2020) or MITOS ( Bernt *et al.*, 2013) and uses these annotations to select the final mitochondrial contig and to ensure the general quality of the sequence.

A Hi-C map for the final assembly was produced using bwa-mem2 ( Vasimuddin *et al.*, 2019) in the Cooler file format ( Abdennur & Mirny, 2020). To assess the assembly metrics, the *k*-mer completeness and QV consensus quality values were calculated in Merqury ( Rhie *et al.*, 2020). This work was done using Nextflow ( Di Tommaso *et al.*, 2017) DSL2 pipelines “sanger-tol/readmapping” ( Surana *et al.*, 2023a) and “sanger-tol/genomenote” ( Surana *et al.*, 2023b). The genome was analysed within the BlobToolKit environment ( Challis *et al.*, 2020) and BUSCO scores ( Manni *et al.*, 2021; Simão *et al.*, 2015) were calculated.

Table 3 contains a list of relevant software tool versions and sources.

**Table 3.** Software tools: versions and sources.

Software tool	Version	Source
BlobToolKit	4.2.1	https://github.com/blobtoolkit/blobtoolkit
BUSCO	5.3.2	https://gitlab.com/ezlab/busco
Hifiasm	0.16.1-r375	https://github.com/chhylp123/hifiasm
HiGlass	1.11.6	https://github.com/higlass/higlass
Merqury	MerquryFK	https://github.com/thegenemyers/MERQURY.FK
MitoHiFi	3	https://github.com/marcelauliano/MitoHiFi
PretextView	0.2	https://github.com/sanger-tol/PretextView
purge_dups	1.2.5	https://github.com/dfguan/purge_dups
sanger-tol/genomenote	v1.0	https://github.com/sanger-tol/genomenote
sanger-tol/readmapping	1.1.0	https://github.com/sanger-tol/readmapping/tree/1.1.0
YaHS	1.2a	https://github.com/c-zhou/yahs

### Genome annotation

The Ensembl Genebuild annotation system ( Aken *et al.*, 2016) was used to generate annotation for the *Diarsia dahlii* assembly (GCA_949775195.1) in Ensembl Rapid Release at the EBI. Annotation was created primarily through alignment of transcriptomic data to the genome, with gap filling via protein-to-genome alignments of a select set of proteins from UniProt ( UniProt Consortium, 2019).

### Wellcome Sanger Institute – Legal and Governance

The materials that have contributed to this genome note have been supplied by a Darwin Tree of Life Partner. The submission of materials by a Darwin Tree of Life Partner is subject to the **‘Darwin Tree of Life Project Sampling Code of Practice’**, which can be found in full on the Darwin Tree of Life website here. By agreeing with and signing up to the Sampling Code of Practice, the Darwin Tree of Life Partner agrees they will meet the legal and ethical requirements and standards set out within this document in respect of all samples acquired for, and supplied to, the Darwin Tree of Life Project.

Further, the Wellcome Sanger Institute employs a process whereby due diligence is carried out proportionate to the nature of the materials themselves, and the circumstances under which they have been/are to be collected and provided for use. The purpose of this is to address and mitigate any potential legal and/or ethical implications of receipt and use of the materials as part of the research project, and to ensure that in doing so we align with best practice wherever possible. The overarching areas of consideration are:

•   Ethical review of provenance and sourcing of the material

•   Legality of collection, transfer and use (national and international)

Each transfer of samples is further undertaken according to a Research Collaboration Agreement or Material Transfer Agreement entered into by the Darwin Tree of Life Partner, Genome Research Limited (operating as the Wellcome Sanger Institute), and in some circumstances other Darwin Tree of Life collaborators.

## Data Availability

European Nucleotide Archive. *Diarsia dahlii*. Accession number PRJEB60641; https://identifiers.org/ena.embl/PRJEB60641 ( Wellcome Sanger Institute, 2023). The genome sequence is released openly for reuse. The *Diarsia dahlii* genome sequencing initiative is part of the Darwin Tree of Life (DToL) project. All raw sequence data and the assembly have been deposited in INSDC databases. Raw data and assembly accession identifiers are reported in Table 1. Members of the Natural History Museum Genome Acquisition Lab are listed here: https://doi.org/10.5281/zenodo.7139035. Members of the Darwin Tree of Life Barcoding collective are listed here: https://doi.org/10.5281/zenodo.4893703. Members of the Wellcome Sanger Institute Tree of Life Management, Samples and Laboratory team are listed here: https://doi.org/10.5281/zenodo.10066175. Members of Wellcome Sanger Institute Scientific Operations: Sequencing Operations are listed here: https://doi.org/10.5281/zenodo.10043364. Members of the Wellcome Sanger Institute Tree of Life Core Informatics team are listed here: https://doi.org/10.5281/zenodo.10066637. Members of the Tree of Life Core Informatics collective are listed here: https://doi.org/10.5281/zenodo.5013541. Members of the Darwin Tree of Life Consortium are listed here: https://doi.org/10.5281/zenodo.4783558.
