# Supplementary material for: The genome sequence of the Barred Chestnut moth, Diarsia dahlii (Hübner, 1813)
Source: Wellcome Open Res. 2024 Jul 5;9:357. [Version 1] doi: 10.12688/wellcomeopenres.22587.1 (PMC11406134; doi:10.12688/wellcomeopenres.22587.1)
Supplement: Peer review history

# Reviewer report: Reviewer response for version 1

**DOI:** 10.21956/wellcomeopenres.24885.r96017

**Published:** 2024-09-16

**Authors:** Jose Martinez

**Affiliations:** 1 University of Florida, Florida, USA

Lees reports a highly complete and well-assembled genome of the Barred Chestnut moth, *Diarsia dahlii* (Hübner, 1813), based on sequencing from a female specimen. The assembly, featuring an exceptionally high BUSCO score, is organized into 32 chromosomal pseudomolecules, including fully recovered Z and W sex chromosomes. This achievement, supported by rigorous methodological approaches, has produced a remarkably detailed and high-quality genomic resource.

 The creation of the dataset is underpinned by a clear and well-structured rationale, with descriptions that are concise and easy to comprehend. The protocols and methodologies are meticulously selected, ensuring both accuracy and reproducibility. The dataset itself is highly standardized, complemented by detailed illustrations, and readily accessible, making it an invaluable resource for a wide range of research applications.

 The author further compared this mitogenome with other specimens and species within the same genus, highlighting its significance in addressing systematic and taxonomic challenges, particularly within the family Noctuidae and related groups.

Are sufficient details of methods and materials provided to allow replication by others?

Yes

Is the rationale for creating the dataset(s) clearly described?

Yes

Are the datasets clearly presented in a useable and accessible format?

Yes

Are the protocols appropriate and is the work technically sound?

Yes

Reviewer Expertise:

Evolutionary Biology, Ecology, IPM, Integrative Biology, Entomology, Genomics

I confirm that I have read this submission and believe that I have an appropriate level of expertise to confirm that it is of an acceptable scientific standard.

# Reviewer report: Reviewer response for version 1

**DOI:** 10.21956/wellcomeopenres.24885.r94049

**Published:** 2024-09-03

**Authors:** Annabel Whibley

**Affiliations:** 1 The University of Auckland, Auckland, Auckland, New Zealand; 2 Grapevine Improvement, Bragato Research Institute, Lincoln, New Zealand

Lees and colleagues describe the sequencing, genome assembly and annotation of a female *Diarsia dahlii* moth (known as the Barred Chestnut moth). The genomic resources generated are of high quality, well documented and use appropriate methods. Public accession links are working. 

**Minor comments:**

 The language in the description of coloration is quite technical- perhaps the authors could consider providing alternative, simpler terms to explain “reniform” and “orbicular” to non-taxonomists in this section, as they later do for “transverse fasciae”: “The moth is pale brownish or reddish with paler yellow reniform and orbicular stigmata (the latter subtended by a black dot) and contrasting slightly darker or greyish transverse fasciae or ‘bars’…”

 Consider “The mitogenome from this genomic assembly” rather than “…the genomic assembly”. Given that this statement precedes description of the assembly itself, it would help to clarify that you are not referring to a different dataset.

 The section on BOLD BIN divergence would benefit from some framing statements about the questions underpinning  these observations.

Are sufficient details of methods and materials provided to allow replication by others?

Yes

Is the rationale for creating the dataset(s) clearly described?

Yes

Are the datasets clearly presented in a useable and accessible format?

Yes

Are the protocols appropriate and is the work technically sound?

Yes

Reviewer Expertise:

Genomics, Evolution, Bioinformatics

I confirm that I have read this submission and believe that I have an appropriate level of expertise to confirm that it is of an acceptable scientific standard.
